# Assessing the Impact of Pomegranate Peel Extract Active Packaging and High Hydrostatic Pressure Processing on Color and Oxidative Stability in Sliced Nitrate/Nitrite-Reduced Iberian Dry-Cured Loins

**DOI:** 10.3390/foods13030360

**Published:** 2024-01-23

**Authors:** Ramón Cava, Luis Ladero, Enrique Riaguas, M. Carmen Vidal-Aragón

**Affiliations:** 1 Universidad de Extremadura, Tradinnoval Research Group, INBIO G+C, Campus Universitario, 10003 Cáceres, Spain; 2 R&D Department, INCARLOPSA, 37770 Guijuelo, Spain; luis.ladero@incarlopsa.es; 3 Universidad de Extremadura, Complejo Universitario Santa Ana, 06200 Almendralejo, Spain; eriaguass@unex.es (E.R.); mcvidal@univsantana.com (M.C.V.-A.)

**Keywords:** pomegranate peel, high-pressure processing, active packaging, instrumental color, TBA-RS, carbonyls, thiols, dry-cured loin

## Abstract

Our study aimed to assess the impact of active packaging with pomegranate peel extract (0.06 mg gallic acid eq./cm^2^) and/or high-pressure treatment (600 MPa, 7 min) on the instrumental color, lipid, and protein oxidation of Iberian dry loins formulated with reduced nitrate/nitrite levels (0, 37.5, and 150 mg/kg) during 100-day refrigerated storage (4 °C). CIE L*a*b* coordinates were measured, and malondialdehyde, carbonyls, and free thiol contents served as markers for lipid and protein oxidation. Active packaging lowered CIE L* (35.4 vs. 34.1) and a* (15.5 vs. 14.5) and increased yellowness (15.6 vs. 16.3) and hue (45.2 vs. 48.4), while pressurization increased CIE L* (33.1 vs. 36.3) and diminished a* values (16.1 vs. 13.9). Ongoing nitrate/nitrite amounts significantly influenced lipid peroxidation, protein carbonyl formation, and free thiol loss. Active packaging and high-pressure processing had varying effects on carbonyl and thiol contents. Neither pressurization nor active packaging impacted malondialdehyde formation. Pressurization enhanced the formation of 4-HNE (503 vs. 697 pg/g). Protein oxidation proved more sensitive to changes, with active packaging offering protection against protein carbonylation (15.4 vs. 14.7 nmol carbonyls/mg protein), while pressurization induced thiol loss (34.3 vs. 28.0 nmol Cys eq./mg protein). This comprehensive understanding provides essential insights for the meat industry, emphasizing the necessity for customized processing conditions to enhance color stability, lipid preservation, and protein integrity in dry-cured loin slices.

## 1. Introduction

Nitrites and nitrates, recognized as key curing agents in the prolonged drying process, are well-established additives in meat products, playing a key role in imparting a distinctive color and a unique flavor profile, controlling lipid oxidation, preventing food spoilage, and ensuring food safety in cured meats [1].

The controversy surrounding the use of nitrite and nitrate as meat-curing agents emerged in the 1950s and 1960s due to their suspected involvement in the formation of N-nitrosamines, potentially carcinogenic to humans. Growing concerns about the health implications of nitrite, coupled with an increased consumer preference for safer, additive-free foods, have led to a shift in regulatory frameworks [1,2,3]. This trend, characterized by concepts such as “all-natural” and “clean-label”, has prompted food safety authorities, exemplified by the European Union’s Regulation No. 1333/2008 amended by Regulations No. 2015/647 and No. 2023/2018, to establish maximum residual amounts for nitrite and nitrate in traditional dry-cured products [4,5,6]. Consequently, the meat industry and researchers face the challenge of developing alternatives to reduce the use of these additives without compromising microbial, sensory characteristics and physicochemical parameters, aligned with evolving public quality demands.

Iberian dry-cured products, recognized as shelf-stable ready-to-eat (RTE) meat foods, owe their stability to low water activity, moderate salt content, nitrite, and moisture levels. However, challenges arise during manufacturing processes, including handling, cutting, slicing, and final packing operations, which may contaminate them with pathogens and spoilage organisms [7,8]. Furthermore, sliced dry-cured products are prone to lipid oxidation, a critical aspect affecting food storage, leading to rancidity issues and meat discoloration. Microbial growth, discoloration, and lipid oxidation are key determinants of the shelf life and consumer acceptance of packaged dry-cured products from Iberian pigs [9,10].

The direct addition of antioxidants to food and packaging technologies, such as vacuum packaging and modified atmosphere packaging (MAP), have been proposed to mitigate the oxidation-related decay that affects both shelf life and product quality. Active packaging, involving the incorporation of additives into packaging materials to release or absorb substances, has shown advantages in terms of antimicrobial and antioxidant activities compared to its direct addition to meat products, as it requires smaller amounts of active compounds, localizes the active compounds on the food surface, prolongs the protection due to migration from film to the food matrix, and maintains a similar food industrial process without introducing new steps to add active compounds [8,11]. The use of active packaging based on olive leaf and rice bran extract combined with high-pressure processing has been evaluated in several publications with sliced dry-cured shoulders [12] and hams [13].

The increasing preference for natural meat products has spurred the search for new additives derived from natural antioxidants and antimicrobials from plant sources to replace artificial antioxidants [8,14]. An economically viable source of natural antioxidants is the by-products of fruit and vegetables [15]. Pomegranate peel (PP) emerges as a valuable by-product, representing 40–50% of the total fruit weight, and is typically discarded. Numerous studies highlighted the high antioxidant activity of pomegranate peel, attributed to substantial amounts of phenolic compounds, including phenolic acids, flavonoids, and hydrolysable tannins [16]. Given the well-known physiological properties of phenolic compounds, encompassing antimicrobial, antiallergenic, anti-inflammatory, and antioxidant activities [17], PP extracts appear to be a promising and economical source of natural antioxidants, preserving and improving the quality of meat products [15,16].

Presently, high hydrostatic pressure (HHP) processing stands out as the most popular non-thermal preservation/processing technology aligning with consumer demands for clean-label products and eco-friendly technologies. This technology extends the shelf life and quality of fresh and processed food products without adding chemical preservatives or additives, having a minimal impact on organoleptic properties [8,18]. HPP processing holds potential as a post-processing decontamination technology for nitrite-free and RTE meat products, ensuring their quality and food safety during storage [8,9,10]. While HPP treatment may induce lipid oxidation, especially in products with no added sulfites or nitrites, affecting the quality of sliced Iberian dry-cured loins [11], the combined effects of active packaging, developed with natural antioxidants such as those from pomegranate peel, and HPP treatment, emerge as an intriguing alternative. This combination could enhance food safety and control lipid oxidation in RTE meat products, potentially requiring lower pressure levels and minimizing the impact on product quality [19]. The application of PP extracts, rich in phenolic compounds, to the development of active packaging is particularly promising due to the resistance of these compounds to HPP [12].

To date, there is a notable gap in the research on the quality changes in dry-cured products treated by active packaging, either individually or combined with HPP, especially in sliced Iberian dry-cured loins with reduced levels of nitrate and nitrite. While Amaro-Blanco et al. [12] and Martillanes et al. [13] explored this in sliced dry-cured shoulders and hams, respectively, our study aims to fill this gap by evaluating the effects of active packaging with pomegranate peel extract and/or high-pressure treatment on the instrumental color, and the lipid and protein oxidation, of Iberian dry loins formulated with reduced levels of nitrate/nitrite over a 100-day refrigerated storage period.

## 2. Materials and Methods

### 2.1. Preparation of Pomegranate Peel Extract (PPE)

Pomegranate fruits (*Punica granatum*, L., Mollar cultivar) were directly harvested from pomegranate trees during the autumn season. Fruits were washed in cold tap water and drained, manually cut, and peeled to separate the seeds and peel. The obtained peel was cut into small pieces and dried at 70 °C for 24 h in a laboratory oven (Memmert GmbH + Co., KG, Schwabach, Germany). After drying, the peel pieces were cooled, powdered using a Grindomix GM 200 knifemill (Retsch GmbH, Haan, Germany), and stored at room temperature until extraction. The PPE was prepared by mixing 100 g of dried peel powder with 900 mL of acetone/water (70/30 *v*/*v*) at 20 °C for 2 h using a rock and roller mixer (Cole-Parmer, Staffordshire, UK). The extract was then filtered through filter paper (Whatman^®^ qualitative filter paper, Grade 1). Subsequently, the acetone solvent was removed using a rotary evaporator (Heidolph, Schwabach, Germany) at 30 °C, and the remaining extract was freeze-dried (SP Scientific, Gardiner, NY, USA) at −20 °C, 0.6 Pa for 72 h. The resulting freeze-dried extract was stored in a light-protected container at −20 °C until use.

### 2.2. Preparation of the Active Packaging

To create the active packages, food-grade polyamide polyethylene packages (20/70) with a thickness of 70 μm and oxygen permeability of 50 cm^3^ mm/m^2^ 24 h atm climate 23 ± 2 °C 75% HR were cut into 11 × 18 cm (198 cm^2^ film surface) bags. All the active packages were prepared the day before packaging the slices of Iberian dry loins. A solution of pomegranate peel extract (372.60 mg/mL total phenolic compounds) was prepared with 1.5 g of the freeze-dried extract dissolved in 5 mL of water:ethanol (3:7 *v*/*v*) to facilitate its application to the internal surface of the bag. Active packaging was prepared under sterile conditions in a laminar flow chamber by dispensing the extract solution at doses of 0.1 mL/198 cm^2^ (equivalent to 0.06 mg total phenolic compounds/cm^2^), spreading 0.05 mL on each side of the bag’s internal surface. The extract solution was applied using an automatic pipette and evenly distributed over the entire surface with a sterile Digralsky spreader. The packages were then dried overnight in a laboratory oven at 25 °C.

### 2.3. Total Phenolic Content (TPC) and Antioxidant Activities Determinations

The antioxidant compounds were analyzed in both the extract solution and the bags. The total phenolic content and antioxidant activities on the surface of the bags were measured just before packaging the samples (2 bags) and after their treatment. For these determinations, five randomly selected sections on the bags were manually cut into 1 cm^2^ squares and extracted by stirring at 25 °C for 30 min (1700 rpm) with 2 mL water:ethanol (3:7 *v*/*v*).

The total phenolic content was determined according to the Folin–Ciocalteau spectrophotometric method [20]. Briefly, 50 µL of sample or gallic acid standard solution (0.5–0.01 mg/mL) was diluted with 450 µL milliQ water and 20 µL Folin–Ciocalteau reagent. After vertexing, 50 µL 20% Na_2_CO_3_ and 450 µL milliQ water were added and incubated at 20 °C for 1 h. The absorbance was measured at 765 nm. 

The 2,2′-azino-bis-3-ethylbenzothiazoline-6-sulfonic acid (ABTS) radical scavenging analysis was conducted spectrophotometrically [20]. Samples and standards (Trolox in 50% MeOH, 0.25 to 1.0 mg/mL) were mixed with ABTS*^+^ radical. The reaction was measured at 734 nm for changes in fluorescence every minute for 15 min.

The 1,1-diphenyl-2-picrylhydrazyl (DPPH) radical scavenging assay was conducted spectrophotometrically [20]. Samples and standards (Trolox, 0.04–0.64 mM) were reacted with DPPH* radical and the changes in absorbance at 515 nm was recorded at 0, 30, and 60 min.

The ferric reducing antioxidant power (FRAP) assay was carried out spectrophotometrically [20]. Samples and standards (FeSO_4_.7H_2_O, 0.023–0.6 mM) were mixed with FRAP reagent and incubated at 37 °C for 30 min. The reaction was measured at 593 nm.

### 2.4. Preparation of Iberian Dry-Cured Loin Samples

Three batches of Iberian dry-cured loins (n = 5) were prepared with different ongoing amounts of potassium nitrate/sodium nitrite (NOx): (a) 150 mg/kg NaNO_2_ + 150 mg/Kg KNO_3_, (b) 37.5 mg/kg NaNO_2_ + 37.5 mg/Kg KNO_3_, and (c) 0 mg/kg NaNO_2_ + 0 mg/Kg KNO_3_, as described previously [21].

At the end of processing, dry-cured loins from the three experimental batches were sliced and randomly distributed into four treatments: 1. Control: dry-cured Iberian loins vacuum packaged; 2. Active packaging (ActPack): dry-cured Iberian loins vacuum packaged in pomegranate peel extract active packaging; 3. Control + HHP: dry-cured Iberian loins vacuum packaged and high-pressure treated; and 4. ActPack + HHP: dry-cured Iberian loins vacuum packaged in pomegranate peel extract active packaging and high-pressure treated. 

Loins were sliced using a gravity-fed food slicer (Mainca, Granollers, Spain) and vacuum packaged just before the pressure treatment. 

**Scheme 1:**
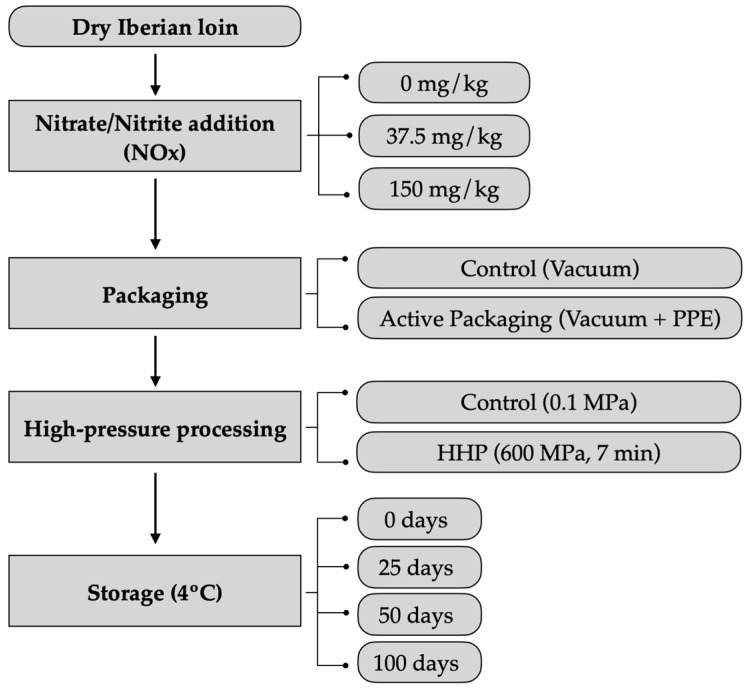
Experimental design.

High-pressure treated samples were pressurized at 600 MPa for 7 min, using water at 10 °C as the pressure-transmitting medium, in a Hyperbaric Wave 6000/55 semi-industrial discontinuous hydrostatic unit (NC Hyperbaric, Burgos, Spain). All samples were stored for 100 days at +4 °C in darkness. Sampling was performed on days 0, 25, 50, and 100 of refrigerated storage. Five packages per treatment were prepared. A total of 240 samples were processed (NOx: 3 levels × HHP: 2 levels × ActPack: 2 levels × Storage: 4 levels × 5 replica) (Scheme 1).

### 2.5. Instrumental Colour and Reflectance Spectrum

The surface color of sliced Iberian dry-cured loins was measured on nine randomly selected locations per sample immediately after opening the package following AMSA recommendations [22] using a Minolta CM-600d colorimeter (Minolta Camera, Osaka, Japan) with a 0.8 cm port/viewing area, a 10° viewing angle, and a D65 illuminant. Before use, a white (CR-A43) and a black tile (CM-A182) were used for instrument calibration. Objective color coordinates CIE L*a*b* were recorded and were used to calculate the psychophysical magnitude hue (H°) (H° = arctan b*/a*). Reflectance spectra were recorded at 10 nm intervals between 360 nm and 740 nm. The cured index (CI) was obtained by the formula R_650 nm_/R_570 nm_.

### 2.6. Thiobarbituric Acid Reactive Substances (TBA-RSs) Determination 

Thiobarbituric acid reactive substances (TBA-RSs) were determined using the method outlined by Lavado and Cava [20]. Loin samples were homogenized in distilled water (1:10 *w*/*v*). Subsequently, 150 µL of the homogenate was combined with 50 µL of 3 N NaOH in a polypropylene tube. The mixture was heated at 60 °C for 30 min. Following this, 250 µL of 6% H_3_PO_4_ and 250 µL of 0.8% thiobarbituric acid were added, and the mixture was heated at 90 °C for 45 min. After incubation, the samples were cooled on ice and extracted with 100 µL of 10% SDS and 600 µL of butanol. Finally, the samples were centrifuged at 3000× *g* for 10 min, and the absorbance of the butanol layer was measured at 532 nm. Quantification was performed using a 1,1,3,3-tetramethoxypropane (TEP) curve (7.2–0.056 µg MDA/mL). The TBA-RS was expressed as mg MDA/kg of the sample.

### 2.7. Quantification of 4-HNE and Saturated Aldehydes

The contents of 4-HNE and saturated aldehydes (pentanal, hexanal, heptenal, octanal, and nonanal) were analyzed by HPLC-FD in dry loins following the formation of their fluorescent derivatives with 1,3-cyclohexanedione, according to Van Hecke et al. [23] with some adaptations described in Lavado and Cava [20]. 

The contents of 4-HNE and saturated aldehydes in homogenized samples were derivatized using cyclohexadione (CHD) derivatizing agent and analyzed via reversed-phase high-performance liquid chromatography (RP-HPLC) using a Shimadzu HPLC system (Shimadzu Co., Kyoto, Japan) equipped with a column oven and a fluorescence detector. An aliquot of 20 µL of the sample was injected into a Shimadzu HPLC equipped with a Cosmosil 5C18-MS-II column (250 mm × 4.6 mm ID, 5 μm, no. 38020-41) (Nacalai Tesque, Inc., Kyoto, Japan) using a linear gradient elution from 0% to 50% over 40 min with aqueous tetrahydrofuran (THF) and maintaining at 50% THF over 10 more min. The flow rate was fixed at 0.8 mL/min and the temperature of the column was kept at 25 °C. The eluted compounds were monitored by fluorescence (λ_exc_: 380 nm; λ_em_: 446 nm). For the identification and quantification of 4-HNE, pentanal, hexanal, heptanal, octanal, and nonanal, standards were used and calibration curves prepared.

### 2.8. Carbonyls from Protein Oxidation Determination

Protein oxidation was assessed by estimating carbonyl groups using the DNPH-based method [24] with the modifications described in Lavado and Cava [20]. In brief, loin samples (1 g) were homogenized at 10,000 rpm for 40 s with 10 mL of chilled 0.15 M KCl. Two aliquots (0.1 mL) of the homogenate were mixed with 1 mL of 10% trichloroacetic acid (TCA) and centrifuged at 5000× *g* for 5 min at 4 °C. Following the removal of the supernatant, 0.4 mL of 5% sodium dodecyl sulphate (SDS) was added, stirred, heated at 100 °C for 10 min, and sonicated at 40 °C for 30 min.

After ultrasonic processing, one sample was treated with 0.8 mL of 0.3% (*w*/*v*) DNPH in 3 M HCl, while the other sample was treated with 0.8 mL of 3 M HCl (referred to as the “blank”). After a 30 min incubation at room temperature, both samples were mixed with 0.4 mL of 40% TCA and centrifuged (5000× *g*, 5 min at 4 °C). Following the removal of the supernatant, pellets were washed with 1 mL of ethanol/ethyl acetate (1/1 *v*/*v*) and centrifuged (10,000× *g*, 5 min, at 4 °C), a process repeated three times. Finally, the pellets were dissolved in 1.5 mL of 6 M guanidine hydrochloride in 20 mM NaH_2_PO_4_ (pH 6.5) and incubated at +4 °C overnight.

Absorbance was spectrophotometrically measured at 370 nm and 280 nm. The protein carbonyl content (nmol/mg protein) was calculated as follows:

(1) $$\mathrm{Carbonyl}\;\mathrm{content}=\frac{{\mathrm{Abs}}_{370}-{\;\mathrm{Abs}}_{370}\left(\mathrm{blank}\right)}{22,000\times \left[{\mathrm{Abs}}_{280}-\left({\mathrm{Abs}}_{370}-{\mathrm{Abs}}_{370}\left(\mathrm{blank}\right)\right)\times 0.43\right]}\times 10^{6}$$

### 2.9. Protein Thiol Determination

The concentration of thiol groups in a 1.5 g loin sample was determined spectrophotometrically after derivatization with Ellman’s reagent, 5,5′-Dithiobis(2-nitrobenzoic acid) (DTNB), following the method described by Martínez et al. [25]. Loin samples (1.5 g) were homogenized (11,600 rpm, 30 s, with ice cooling) with 12.5 mL of 0.05 M 2-(N-morpholino) ethane sulfonic acid buffer (MES) (pH 5.8). An aliquot of 0.5 mL of the homogenate was mixed with 1.5 mL of 5% SDS in 0.10 M tris(hydroxymethyl)-aminomethane (TRIS) buffer (pH 8.0) and heated at 80 °C for 30 min. Samples were then centrifuged (3000× *g*, 20 min), and the supernatants were filtered using a syringe filter (PTFE, 0.45 µm).

For thiol concentration determination, an aliquot of 0.05 mL of the filtrate was mixed with 0.2 mL of 5% SDS/TRIS buffer (pH 8.0) and 0.05 mL of 10 mM DTNB in 0.10 M TRIS buffer (pH 8.0). The absorbance was measured spectrophotometrically at 412 nm before the addition of the DTNB reagent (Abs_412 nm before_) and after incubation for 30 min in the dark (Abs_412 nm after_). Thiol concentration was calculated using the formula:

(2) $${\mathrm{Corr}}_{\mathrm{Abs}\;412}={\;\mathrm{Abs}}_{412\;\mathrm{nm}\;\mathrm{after}}-{\mathrm{Abs}}_{412\;\mathrm{nm}\;\mathrm{before}}-{\mathrm{Abs}}_{412\;\mathrm{nm}\;\mathrm{blank}}$$

A calibration standard of L-Cys, diluted in 5% SDS/TRIS buffer (pH 8.0) at concentrations ranging from 32.25 µM to 1000 µM, was utilized for thiol quantification. Protein concentration was determined using the Pierce Rapid Gold BCA protein assay reagent (Thermo Fisher Scientific Inc., Waltham, MA, USA), following the manufacturer’s instructions. Spectrophotometric absorbance was measured at 480 nm, using bovine serum albumin (BSA) diluted in 5% SDS/TRIS buffer (pH 8.0) as an external calibration standard spanning concentrations from 0.3125 g/L to 2.5 g/L. The measurements were conducted using a Multiskan Go spectrophotometer (Thermo Fisher Scientific, Vanta, Finland). Results were expressed as nmol Cys eq./mg protein.

### 2.10. Statistical Analysis

The effect of time of storage on antioxidant compounds and activities in the films was analyzed via one-way analysis of variance (ANOVA) within the statistical software Jamovi (https://www.jamovi.org (accessed on 1 November 2023)) (Version 2.0.0.0). Tukey’s test was used to compare differences among mean values when ANOVA showed significance (*p* < 0.05). Means and standard error of the mean were reported.

The data were analyzed utilizing the Mixed Linear Model procedure (GAMLj module, https://gamlj.github.io/ (accessed on 1 November 2023)) within the statistical software Jamovi. A repeated-measure mixed-model analysis was used, encompassing ongoing amounts of nitrate/nitrite, active packaging, high-pressure treatment, and time of storage as fixed effects, with the loin sample considered as the random effect. Upon detecting significant effects within the treatment groups (*p* < 0.05), a Bonferroni test was conducted for mean comparisons. Results were reported in terms of mean values and standard error of the mean (SEM).

## 3. Results and Discussion

### 3.1. Active Packaging Antioxidant Characteristics

The PPE exhibited an elevated content of phenolic compounds (373 ± 1.1 mg gallic acid eq./g freeze-dried extract), along with significant antioxidant activity (FRAP: 594 ± 27.6 μg Fe^2+^/g freeze-dried extract) and antiradical activities (878 ± 52.1 and 450 ± 63.7 mg Trolox eq./g freeze-dried extract for ABTS and DPPH, respectively).

The antiradical activities in the bags just before packaging were 0.203 and 0.066 mg equivalents Trolox/cm^2^ for ABTS and DPPH activities and the antioxidant activity was 0.096 μg Fe^2+^/cm^2^ (Table 1). The concentration of phenolic compounds and antioxidant activities in the active packaging formulated with PPE exhibited a progressive decline throughout the storage period. This decrease in phenolic compounds, along with the reduction in ABTS, DPPH, and FRAP activities, could be ascribed to both the depletion while functioning as antioxidants and the subsequent transfer to the slices of cured loin.

### 3.2. Instrumental Colour Parameters

The ongoing amounts of NOx, high-pressure processing, and active packaging significantly affected the instrumental color parameters during the refrigerated storage (Table 2).

The inclusion, reduction, or removal of NOx had an impact on the instrumental color parameters of packed loin slices, namely CIE L*, b*, hue, and the curing index. The reduction in (37.5 mg NOx/kg) and removal of NOx (0 mg NOx/kg) resulted in a significant enhancement of lightness (CIE L*) and yellowness (CIE b*), consequently increasing the final H° value. The reduction in the ongoing amounts of NOx (37.5 mg NOx/kg) did not produce any discernible alteration in the CI value compared to 150 mg NOx/kg dry-cured loin slices; however, removal of NOx (0 mg NOx/kg) led to a significant decrease in the cured index. 

These results contrast with the absence of significant differences between dry-cured loins formulated with reduced ongoing amounts of nitrate and nitrite (150–75 mg/kg), as reported by Belloch et al. [26]. Additionally, they differ from the substantially lower CIE L* values observed in dry-cured loins in which curing salts (140 mg NaNO_2_/kg) were replaced by sea salt [27]. However, paprika addition led to a reduction in lightness, which suggests that the lower CIE L* values in the loin sliced from the 150 NOx batch could be related to higher amounts of nitrifying salts due to the contribution of paprika to the overall nitrate content [26].

On the other hand, 0 mg NOx/kg sliced dry loins showed lower CIE a* values than their cured counterparts (37.5 and 150 mg NOx/kg), consistent with the absence of differences between dry-cured loins with reduced ongoing amounts of NOx, as reported by Belloch et al. [26]. Moreover, the 37.5 and 0 NOx loin slices exhibited higher CIE b* and H° values than the 150 NOx counterparts. Higher H° values indicate lower redness and a less stable red color [28]. This fact could be attributed to the formation of nitrosylmyoglobin in dry-cured loins formulated with nitrate and nitrite, and Zn-protoporphyrin IX in uncured dry loins [29,30], as well as the potential interference of paprika in color parameters.

Pressurization caused an increase in CIE L* and H° values, accompanied by a decrease in CIE a* and CI. Similar findings have been reported by various authors in dry-cured hams (500–600 MPa, 5–6 min) [31,32,33], dry-cured loin (300–400 MPa, 10 min) [34], and dry-cured Iberian loin (600 MPa, 8 min) [10]. This higher lightness value is related to the increase in the ratio of reflected/absorbed light, resulting from the rearrangement of proteins after the high-pressure-induced denaturation of myofibrillar proteins [32,35,36].

Furthermore, pressurization produced a decrease in CIE a* values, which is in accordance with the lower redness values observed in pressurized dry-cured loins and dry-cured hams [10,32,34], but not with results previously reported in sliced dry-cured shoulders [12] and hams [13]. The decrease in redness has been attributed to the partial denaturation of the globin part of nitrosyl-myoglobin and the formation of nitrosyl hemochromogen [32]. In other studies, the effect of pressurization has been reported to increase or not change redness in dry-cured hams [31]. These differences could be explained by the diverse characteristics of the raw materials, the processing conditions before and during HHP treatment, and the type of storage. Furthermore, as anticipated from the CIE a* values, lower H° values were observed in non-pressurized loin slices, suggesting a more pronounced and stable red color.

Regarding the influence of active packaging on instrumental color coordinates, loin slices packaged with the active material displayed reduced CIE L* and a* values, leading to darker and less red loin slices. This was accompanied by an increase in b* and a concurrent rise in H° value. The CI remained unaffected by active packaging. Our findings contrast with those of Amaro-Blanco et al. [12] and Martillanes et al. [13], who reported no effect when using olive leaf and rice bran extract active packaging, with or without high-pressure treatment, on the instrumental color coordinates of dry-cured shoulders and hams during 150 days of storage.

Storage had a discernible effect on CIE L*, a*, and b* values, as well as the CI, resulting in a gradual decrease in these values throughout the 100-day period of refrigerated storage. This trend suggests a darkening of the dry loin slices and a diminishing redness, as has been reported in previous studies [10,12,13].

### 3.3. Lipid Oxidation

The ongoing amounts of nitrate/nitrite had a noticeable influence on lipid peroxidation (Table 3). 

No significant differences in TBA-RS were observed between samples containing 150 mg/kg and those with 0 mg/kg, both of which exhibited significantly higher values compared to the 37.5 mg/kg samples. Contrary to our expectations, our results do not support an antioxidant effect mitigating the formation of malondialdehyde, as has been reported by other authors [37,38].

The limitation on lipid oxidation caused by nitrite arises from various mechanisms, including the stabilization of heme iron and the chelation of other metal ions, which are prominent pro-oxidants in meat products. Bonifacie et al. [38] demonstrated in cured meat models with reduced doses of sodium nitrite (0–120 mg/kg) that the absence of nitrite led to an increase in free iron, a known promoter of oxidative processes through the Fenton reaction. Nitric oxide, generated from nitrite, readily reacts with oxygen, swiftly sequestering oxygen and other reactive oxygen species. It also reacts with lipid radicals, inhibiting radical chain reactions of oxidation. However, the interplay of low oxygen partial pressure and low pH (5.0–6.0) in cured meats creates conditions that amplify the formation of both nitric oxide and superoxide. These conditions are key precursors of peroxynitrite [39]. Consequently, in cured meats with high amounts of both curing salts, such as the 150 NOx batch, peroxynitrite becomes involved in the production of reactive species that can induce lipid oxidation [40].

Pressurization did not induce changes in malondialdehyde formation when compared to non-pressurized samples. TBA-RS values were statistically equivalent between pressurized and non-pressurized samples, indicating that high-pressure treatment did not affect lipid peroxidation. These results are in line with those reported in dry-cured shoulders [12] and hams [13] in which no effect of pressurization on the levels of malondialdehyde was found. However, previous research reflects a pro-oxidant effect of pressurization with increased levels of TBA-RS in dry-cured loins during short cold storage [10], while other authors reported reductions in TBA-RS in pressurized dry-cured meats [31,34]. The role of high pressure on lipid oxidation has been the subject of numerous studies, revealing diverse effects contingent upon the type of meat product (fresh, cooked, dried, cured) and pressurization conditions. Despite variations among experiments, it is generally acknowledged that the release of iron (Fe) from the heme group of myoglobin, mediated by high pressure, and the involvement of this Fe in the Fenton reaction, are directly implicated, among other factors, in the amplification of lipid oxidation. However, it is noteworthy that this release is influenced by the stabilization of Fe by nitric oxide in cured meats [41]. 

The implementation of active packaging did not exert an antioxidant effect against lipid peroxidation. Loin slices under active packaging did not show a significant reduction in their TBA-RS values compared to their control counterparts. Our results align with those previously obtained in dry-cured ham and shoulders packaged with active packaging. It is crucial to acknowledge the distinct differences between the aforementioned studies and the present investigation, particularly in terms of the type of packaging utilized (antioxidant activity extract, dosage, application method) and the unique characteristics of the packaged products in each experiment [12,13].

The impact of storage on lipid oxidation became increasingly evident over time. Contrary to what was expected, TBA-RS values significantly decreased during the storage period. Specifically, TBA-RS values on day 0 exceeded those on day 25, which, in turn, were higher than those on day 50. Surprisingly, no significant difference emerged between TBA-RS values on days 50 and 100. These observations suggest a dynamic evolution of lipid oxidation during storage, where formed MDA may further react with amino acids, sugars, and nitrite, contributing to the decline in TBA-RS values over time [42,43].

Nitrate/nitrite in loins significantly affected the contents of 4-HNE and heptanal, while the contents of the remaining saturated aldehydes were unaffected (Table 3). The removal of nitrate/nitrite from the formulation of Iberian loins caused a significant increase in 4-HNE and heptanal contents compared to loins cured with ongoing amounts of 37.5 and 150 mg NOx/kg. Reduced ongoing amount of nitrate/nitrite (37.5 mg NOx/kg) had a different effect on the 4-HNE and heptanal contents. 

While the 4-HNE content significantly decreased compared to the uncured counterparts (0 mg/kg), the heptanal content did not differ from the other two remaining product batches (0 and 150 mg/kg). 4-HNE, a major secondary oxidation product resulting from the lipid peroxidation of omega-6 fatty acids, has been recognized as a marker of food quality in meat products. This is attributed to 4-HNE’s ability to form adducts with histidine, promoting myoglobin redox instability that, in turn, enhances lipid oxidation [44]. Moreover, extensive research has been conducted on 4-HNE, revealing its significant role in the pathogenesis of various diseases. It is regarded as one of the principal toxic by-products generated from lipid peroxides. As such, 4-HNE possesses a substantial capacity to engage in covalent adduct formation with multiple biological molecules. This capability may elucidate its involvement in numerous pathological processes, including metabolic and neurodegenerative diseases, as well as certain types of cancer [44,45].

The response to pressurization treatment resulted in varied concentrations of saturated aldehydes and 4-HNE, while pentanal, hexanal, heptanal, and octanal contents remained unaffected. However, HHP led to an increase in the levels of 4-HNE and nonanal in dry loin slices when compared to untreated samples. The application of active packaging had no influence on the concentrations of 4-HNE and saturated aldehydes in Iberian loin, in accordance with the observed trend for TBA-RS.

Storage conditions resulted in a reduction in the concentration of both 4-HNE and saturated aldehydes. Notably, this change reached statistical significance only for pentanal, hexanal, and octanal, while it did not impact the levels of 4-HNE, heptanal, or nonanal.

### 3.4. Protein Oxidation

Table 4 shows that the ongoing amounts of nitrate/nitrite significantly influenced protein carbonylation and thiol loss. In contrast to lipid oxidation, the presence of added nitrate/nitrite significantly reduced the content of carbonyls, reflecting a protective role against protein carbonylation in sliced dried loin. Dry-cured loin slices with 150 mg NOx/kg and 37.5 mg NOx/kg had significantly lower carbonyl contents compared to their uncured counterparts (0 mg NOx/kg). Nitrite has been ascribed various roles, including as a pro-oxidant and antioxidant, and has no effect on protein oxidation [38,46,47,48,49]. Despite its common use in meat-based products, the impact of nitrite on protein oxidation in dry-cured meats remains a subject of controversy. The conflicting results may be attributed to variations in meat product type, nitrite levels, experimental conditions, and other factors. Several hypotheses exist regarding the mechanisms preventing protein oxidation, including nitrite’s ability to sequester oxygen, chelate metals, and inhibit their pro-oxidant actions [38].

Pressurization did not impact carbonyl formation, indicating a negligible effect of high pressure on protein carbonylation phenomena. These results align with those reported in dry-cured Iberian hams after 150 days of storage and dry-cured loins after 90 days of storage [7,12]. The low moisture content and Aw of dried meat products may limit protein oxidation [50]. However, conflicting results have been observed in treated dry-cured loins. Cava et al. [10] reported higher carbonyl values in pressurized loins after 60 days of storage, in contrast to the decreased carbonyl contents reported in HHP-treated loins after 60 days [48]. The lack of consensus in the literature is likely attributable to differences in the types of meat products treated, as well as the conditions of pressure treatment, packaging, and storage [41,50].

In contrast, the carbonyl content was significantly lowered by active packaging, indicating a protective effect against protein oxidation of dry loin slices. Our results demonstrate the effectiveness of PPE active packaging in controlling protein carbonylation. These findings contrast with previous studies assessing active packaging containing olive leaves and rice bran extracts in sliced dry-cured shoulders and hams, where no discernible effects on the carbonyl formation were observed [12,13]. The variations in the effectiveness of different active packaging on carbonyl formation may be linked to the characteristics of the packaged products (such as the initial oxidation level, fat content, and level of nitrifying salts) and the attributes of the extracts (including the incorporation level of the antioxidant extract, the antioxidant activity, and the stability of the active compounds) used in the production of the active packaging.

Finally, the storage time slightly increased the accumulation of protein carbonylation products. A gradual rise in carbonyl contents occurred within the first 50 days of storage, yet without significant deviation from the initial levels. However, at day 100, the amounts of carbonyls were significantly higher compared to the initial concentrations at the onset of the experiment.

In the same sense as described for the carbonyl content, 150 mg NOx/kg protected the proteins from the loss of thiols compared to 37.5 or 0 mg NOx/kg. No differences in the thiol content were observed between loin samples with reduced ongoing amounts of nitrate/nitrite (37.5 mg NOx/kg) and without added nitrate/nitrite (0 mg NOx/kg). The loss of free SH groups is associated with protein oxidation in meat products [51]. Previous works differ from our findings, which described that higher amounts of NaNO_2_ led to decreased contents of SH groups in raw porcine patties and cooked sausages [46,49]. In this sense, it was reported in cysteine and myoglobin model systems that the ongoing nitrite concentration of 500 mg/kg resulted in lesser thiol groups, probably due to the formation of S-Nitrosothiol groups [52].

It is worth noting that pressurization treatment caused a significant decrease in the thiol content of the cured Iberian loin samples. Similarly, decreased thiols in HHP-treated samples were found in dry-cured loins [9,10]. According to Guyon et al. [51], irreversible modifications induced by HHP are produced at 500 MPa. Consequently, thiol oxidation involves the formation of disulfide bridges and the formation of compounds with irreversible bonds, resulting from protein rearrangement and modification of amino acid chains [50] and HHP-induced protein denaturation [53]. Additionally, thiol groups are highly susceptible to oxidation and are known to function as radical scavengers, protecting proteins and valuable amino acids against oxidative damage [54]. Protein carbonylation is commonly considered to occur after thiol oxidation. Consequently, it serves as an indicator of heightened protein oxidation conditions [55].

Active packaging had no impact on thiol concentration. However, thiols displayed an opposite behavior compared to carbonyls during storage. As the storage time increased, there was a notable reduction (~24%) in thiol content from days 0 to 100 of refrigerated storage, with statistically significant differences observed across all sampling days. 

## 4. Conclusions

Our findings emphasize the intricate complexity of simultaneously incorporating various preservation technologies in the packaging of both cured and non-cured dry loins to preserve color and ensure oxidative stability during refrigerated storage. The manipulation of ongoing amounts of curing salts in sliced dry loin distinctly influenced color attributes. High-pressure processing and active packaging, utilizing a pomegranate peel extract, impacted CIE L*, a*, and b* differently, depending on the technology applied. This resulted in either darkening or increased lightness, as well as a loss of redness—alterations that collectively impacted the color profile of the sliced dry loins.

The presence of nitrate/nitrite significantly influenced the extent of lipid peroxidation and the formation of protein carbonyls, as well as the loss of free thiols. The use of active packaging and high-pressure processing had a different effect on the contents of carbonyls and thiols. Neither pressurization nor active packaging had any impact on malondialdehyde formation. However, protein oxidation was more susceptible to change depending on the studied factor, with active packaging providing protection against protein carbonylation, and pressurization inducing the loss of thiols.

The extensive study presented here provides a crucial insight into conditions required to optimize color stability, lipid preservation, and protein integrity in the processing of dry-cured loin slices, using an alternative and more natural method of packaging.

## Figures and Tables

**Table 1:** Total phenolic compounds and antioxidant activities measured on the surface of the bags with pomegranate peel extract (PPE) used in the active packaging.

		Antioxidant Compounds	Antioxidant Activity		
		Total Phenolic Content ^1^	ABTS ^2^	DPPH ^2^	FRAP ^3^
Before packaging ♣		0.056 a	0.203 a	0.066 a	0.096 a
Time of storage (days)	0	0.004 b	0.028 c	0.036 b	0.017 b
	25	0.002 b	0.030 bc	0.020 b	0.010 b
	50	0.003 b	0.052 bc	0.023 b	0.016 b
	100	0.004 b	0.055 b	0.029 b	0.011 b
SEM		0.0008	0.0025	0.0015	0.0015
Sig.		***	***	***	***

♣ Indicates the active packages with the PPE just before dry-cured loin packaging. ^1^ mg gallic acid eq./cm^2^, ^2^ mg equivalents Trolox/cm^2^, ^3^ μg Fe^2+^/cm^2^. ***: *p* < 0.001. a,b,c: Means within the same effect with different superscripts differ significantly (Tukey test *p* < 0.05).

**Table 2:** Effect of nitrate and nitrite ongoing amounts, active packaging, HHP treatment, and time of refrigerated storage on instrumental color parameters (CIE L*a*b*), psychophysical magnitude hue (H°), and cured index of sliced Iberian dry-cured loin.

Effects		CIE L*	CIE a*	CIE b*	Hue	CI
NO_3_^−^/NO_2_^−^ added	0 mg/kg	34.4 a	14.6	16.7 a	48.9 a	2.8 b
	37.5 mg/kg	36.1 a	15.3	16.4 a	47.0 ab	3.2 a
	150 mg/kg	33.7 b	15.1	14.8 b	44.6 b	3.2 a
HHP treatment	Control	33.1	16.1	15.8	44.5	3.3
	HHP-treated	36.3	13.9	16.1	49.2	2.8
Active packaging	Control	35.3	15.5	15.6	45.2	3.1
	ActPack	34.1	14.5	16.3	48.4	3.0
Time of storage (days)	0	37.0 a	16.2 a	16.4 a	45.2	3.4 a
	25	35.2 ab	15.4 ab	16.1 ab	46.3	3.2 a
	50	33.5 b	15.1 b	16.6 a	47.7	3.1 a
	100	33.1 b	13.2 c	14.8 b	48.1	2.7 b
SEM		0.20	0.13	0.12	0.01	0.03
Sig. NOx		*	n.s.	***	***	***
Sig. HHP		***	***	n.s.	***	***
Sig. ActPack		***	***	**	***	n.s.
Sig. Storage		***	***	***	n.s.	***

n.s.: *p* > 0.05, *: *p* < 0.05, **: *p* < 0.01; ***: *p* < 0.001. a,b,c: Means within the same effect with different superscripts differ significantly (Bonferroni test, *p* < 0.05).

**Table 3:** Effect of nitrate and nitrite ongoing amounts, active packaging, HHP treatment, and time of refrigerated storage on lipid oxidation markers (TBA-RS, 4-HNE and saturated aldehydes).

Effects		TBA-RS ^1^	4-HNE ^2^	C5-al ^2^	C6-al ^2^	C7-al ^2^	C8-al ^2^	C9-al ^2^
NO_3_^−^/NO_2_^−^ added	0 mg/kg	3.99 a	920 a	53	65	306 a	11	23
	37.5 mg/kg	3.01 b	441 b	37	77	286 ab	12	20
	150 mg/kg	3.95 a	439 b	40	78	266 b	11	21
HHP treatment	Control	3.71	503	46	75	280	11	19
	HHP-treated	3.59	697	41	72	293	12	24
Active packaging	Control	3.70	648	45	76	286	12	23
	ActPack	3.60	551	42	71	286	11	21
Time of storage (days)	0	4.18 a	627	52	94	289	13	21
	25	3.74 ab	--	--	--	--	--	--
	50	3.36 b	--	--	--	--	--	--
	100	3.32 b	573	35	53	283	10	22
SEM		0.20	39.3	3.5	4.1	5.2	0.5	1.3
Sig. NOx		*	***	n.s.	n.s.	**	n.s.	n.s.
Sig. HHP		n.s.	**	n.s.	n.s.	n.s.	n.s.	*
Sig. ActPack		n.s.	n.s.	n.s.	n.s.	n.s.	n.s.	n.s.
Sig. Storage		***	n.s.	*	***	n.s.	**	n.s.

^1^: mg MDA/kg; ^2^: pg/g. n.s.: *p* > 0.05, *: *p* < 0.05, **: *p* < 0.01; ***: *p* < 0.001. a,b: Means within the same effect with different superscripts differ significantly (Bonferroni test, *p* < 0.05).

**Table 4:** Effect of nitrate and nitrite ongoing amounts, active packaging, HHP treatment, and time of refrigerated storage on carbonyls and thiols of sliced Iberian dry-cured loin.

Effects		Carbonyls ^1^	Thiols ^2^
NO_3_^−^/NO_2_^−^ added	0 mg/kg	16.4 a	30.7 b
	37.5 mg/kg	14.4 b	31.5 ab
	150 mg/kg	14.4 b	32.6 a
HHP treatment	Control	14.9	34.3
	HHP-treated	15.3	28.9
Active packaging	Control	15.4	31.6
	ActPack	14.7	31.6
Time of storage (days)	0	14.6 b	36.4 a
	25	14.7 ab	32.0 b
	50	15.0 ab	30.1 bc
	100	16.0 a	27.9 c
SEM		0.05	0.16
Sig. NOx		***	*
Sig. HHP		n.s.	***
Sig. ActPack		*	n.s.
Sig. Storage		***	***

^1^: nmol carbonyls/mg protein; ^2^: nmol Cys eq./mg protein. n.s.: *p* > 0.05, *: *p* < 0.05, ***: *p* < 0.001. a,b,c: Means within the same effect with different superscripts differ significantly (Bonferroni test, *p* < 0.05).

## Data Availability

The data that support the findings of this study are openly available in Mendeley Data at http://doi.org/10.17632/svc266z5y5.1, (accessed on 22 January 2024).
